# Immune Response to COVID-19 During Pregnancy

**DOI:** 10.3389/fimmu.2021.675476

**Published:** 2021-05-03

**Authors:** Ge Chen, Qiuyue Liao, Jihui Ai, Bin Yang, Hualin Bai, Jing Chen, Fengyuan Liu, Yang Cao, Haiyi Liu, Kezhen Li

**Affiliations:** ^1^Department of Obstetrics and Gynecology, Tongji Hospital, Tongji Medical College, Huazhong University of Science and Technology, Wuhan, China; ^2^Biological Sciences Greenhouse, College of Art & Sciences, The Ohio State University, Columbus, OH, United States; ^3^Department of Hematology, Tongji Hospital, Tongji Medical College, Huazhong University of Science and Technology, Wuhan, China

**Keywords:** COVID-19, pregnant women, immune response, lymphocyte subtypes, cytokines

## Abstract

Pregnant women are generally more susceptible to viral infection. Although the impact of SARS-CoV-2 on pregnant women remains to be determined, evidence indicates that risks of adverse clinical outcomes are similar in pregnancy to the general population. Here we analyzed clinical symptoms and outcomes of 20 pregnant and 299 reproductive-aged non-pregnant female COVID-19 patients who were hospitalized during the same period. Laboratory measurements were compared among mild cases and healthy pregnant women. Our study found that pregnant patients showed enhanced innate immune response evident by higher neutrophils and C-reactive protein. Cytokines, chemokines, and growth factors (CCGFs) profiles from 11 pregnant and 4 non-pregnant COVID-19 patients and 10 healthy pregnant female patients, and lymphocyte subsets analysis of 7 pregnant patients and 19 non-pregnant patients, indicate suppressed cytokine storm and potential enhanced CD8+ T cell and NK cell activity in pregnant patients with COVID-19, which may be essential in contributing to the unique anti-SARS-CoV-2 response in pregnancy.

## Introduction

Since December 2019, coronavirus disease 2019 (COVID-19), caused by severe acute respiratory syndrome coronavirus 2 (SARS-CoV-2), has been spreading rapidly worldwide. As of March 3, 2021, a total of 115,302,067 COVID-19 cases had been diagnosed globally, with a cumulative death toll of 2,560,638 (1).

SARS-CoV-2 enters the cell *via* the angiotensin-converting enzyme type 2 (ACE-2) receptors, which mainly expressed on pulmonary epithelial cells, but also on lymphocytes and other cell types. The spread of the virus among cells triggers both innate and adaptive immune responses, which then causes the production of large amounts of pro-inflammatory cytokines and chemokines (2, 3). In some patients, the activation is massive and develops into a “cytokine storm,” which induces severe inflammatory responses and causing tissue damage or even death (4, 5). Although all age groups are generally susceptible, the susceptibility and clinical outcomes under SARS-CoV-2 are highly heterogeneous due to differences in immune responses of the hosts.

During the COVID-19 pandemic, there was additional concern surrounding pregnant women due to their unique immunological status, which requires adaptions to ensure tolerance to the fetus while preserving immunoprotective functions. Immune states during pregnancy are often characterized by alterations in the cellular composition and the functions of immune cells. T cell-mediated immunity and humoral responses are suppressed in normal pregnancy particularly during the third trimester (6–8). Elevated IL-4, IL-10 and reduced IL-2, IFN-γ production in peripheral blood mononuclear cells (PBMCs) (9, 10) are also observed in pregnancy. It was reported that influenza A virus infection during pregnancy exaggerated the inflammatory response with high levels of pro-inflammatory and anti-inflammatory cytokines (11, 12). Therefore, immune characteristics and clinical outcomes of pregnant women are expected to be different to non-pregnant women with COVID-19 infections.

This heterogeneity has been reported in previous studies indicating that pregnant women are at increased risk of morbidity and mortality from respiratory viral infections, including influenza A virus, severe acute respiratory syndrome associated coronavirus 1 (SARS-CoV-1), and Middle East respiratory syndrome associated coronavirus (MERS-CoV) (13, 14). However, regarding the SARS-CoV-2 infection, studies have shown that pregnant women do not have a greater risk of death than non-pregnant COVID-19 patients (15–17). The largest dataset available so far was published by the Centers for Disease Control and Prevention (CDC), which included 8,207 pregnant women with COVID-19 and indicated that they were more likely to be hospitalized and had increased rates of ICU and mechanical ventilation admissions compared with non-pregnant women with COVID-19, but their risk for death is similar (18). The relatively low risk of death has been attributed to immunological and hormonal factors (19, 20). However, the protective effects are mainly based on speculation, as few studies have investigated the immunological features and underling mechanisms of pregnant patients suffering from COVID-19.

Therefore, we designed this study to compare the clinical and immunological features between pregnant and non-pregnant COVID-19 patients. These findings may help expand our understanding of the immune system’s response to SARS-CoV-2 infection and explore its potential protective mechanisms in pregnant COVID-19 patients, and propose reasonable options of care and treatment.

## Materials and Methods

### Participants and Data Collection

**Figure 1:**
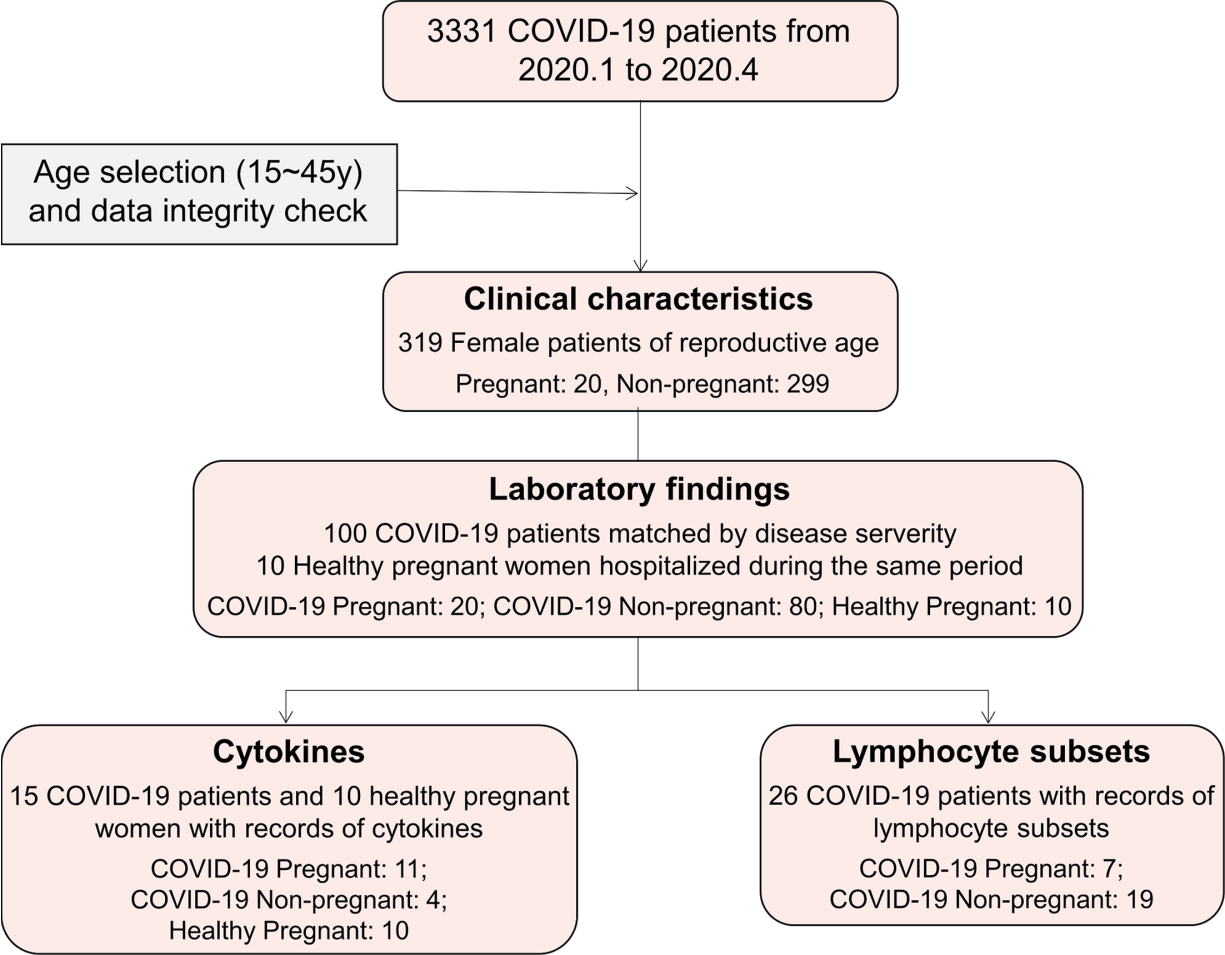
Flowchart of patient recruitment and data analysis procedure. A total of 3,331 COVID-19 patients from Tongji Hospital were screened and a total of 319 reproductive-aged female patients with complete medical recording were enrolled. Patients were grouped as pregnant (n = 20) and non-pregnant (n = 299), and compared by clinical characteristics, laboratory findings, and cytokines. Ten healthy pregnant women were included as control.

In this retrospective single-center study, 20 pregnant women and 299 non-pregnant women of reproductive age with confirmed COVID-19 infection (according to the 7th edition of China’s Diagnosis and Treatment Protocol for Novel Coronavirus Pneumonia) were recruited from January 19 to April 20, 2020 from Tongji Hospital in Wuhan, China. The participants’ demographic data, medical history, chronic disease history, laboratory findings, chest computed tomography (CT) scan findings, and outcomes were reviewed from the patient database. Ten healthy pregnant women hospitalized during the same period were also included as a control group. The flowchart of study design and data analysis was shown in **Figure 1**.

### Definitions

All of the enrolled patients had confirmed COVID-19 according to the Diagnosis and Treatment Protocol for Novel Coronavirus Pneumonia (7th edition). In brief, a patient is diagnosed with COVID-19 if the results of the SARS-CoV-2 RNA or specific antibody test is positive. During their hospital stay, patients are categorized as (1) mild cases if they have mild clinical symptoms with or without typical CT imaging of viral pneumonia; (2) severe cases if their oxygen saturation is ≤93% at rest, they have respiratory distress with a respiration rate (RR) <30 times/min, or their arterial partial pressure of oxygen (PaO2)/oxygen absorption concentration (FiO2) is ≤300 mmHg; or (3) critical cases if the patient suffers from respiratory failure requiring mechanical ventilation, shock, or organ failure requiring intensive care.

Abnormal hepatic function was defined as the alanine aminotransferase level >66 U/L. Abnormal renal function was defined as having a blood creatinine level >84 µmol/L or a blood urea nitrogen concentration >7.5 mmol/L.

Lymphopenia was defined as peripheral blood lymphocyte count <1 × 10^9^/L.

Patients can be discharged if they meet the following conditions specified in the 7^th^ edition of the Diagnosis and Treatment Protocol for Novel Coronavirus Pneumonia: 1. Body temperature has returned to normal and remained normal for more than 3 days; 2. Respiratory symptoms have improved significantly; 3. CT scan shows significant improvement in acute exudative lesions; and 4. Two consecutive sputum and nasopharyngeal swabs, and other respiratory tract specimens have tested negative for nucleic acid (sampling time interval of at least 24 h). Discharge time was defined as the first day the patient met the discharge criteria.

### Blood Specimen Collection and Cytokine Assessment

For the cytokine assays, plasma samples from pregnant patients and non-pregnant patients were collected from the specimen bank at Tongji Hospital. The concentrations of 48 cytokines were measured using the 152 Bio-Plex Pro Human Cytokine Screening Panel (Bio-Rad) as reported previously (21). Samples, standards, blank, and controls were added and followed by detection antibodies. Raw data were generated using the Bio-Plex Manager software. Concentrations below the detection range were considered as zero. Concentrations above the detection range were converted to the highest value of the standard curve. All cytokines were expressed as pg/ml and extrapolated from the standard curves (individual for each cytokine).

### Statistical Analysis

We performed statistical analyses using SPSS version 26.0 (IBM) and R version 3.6.0. Continuous variables were described as median (interquartile range, IQR), and categorical variables were described as number (percentage). For comparison of continuous variables, we used the Mann-Whitney U test for two groups and the Kruskal-Wallis H test for three groups. Pairwise comparison of the Kruskal-Wallis H test was further used to detect the statistic difference between every two groups. The Chi-squared test and Fisher’s exact test (when the theoretical frequency was less than 5 or the sample size is less than 40) were used to compare the categorical variables. This study was approved by the Ethical Committee of Tongji Hospital, Tongji Medical College, Huazhong University of Science and Technology (ID: TJ-IRB2020401). The participants provided their written informed consent to participate in this study.

## Results

### Demographic and Clinical Characteristics of Pregnant Patients and Non-Pregnant Female Patients

Between January 19 to April 20, 2020, 20 pregnant and 299 non-pregnant female COVID-19 patients (from 15 to 45 years old) were admitted to Tongji Hospital. The clinical symptoms were listed in **Table 1**. No significant difference was found in comorbidities, onset symptoms, and complications between pregnant patients and non-pregnant counterparts (**Table 1**). In our cohort, none of pregnant patients developed severe COVID-19 symptoms, which was significantly lower than non-pregnant group (**Table 1**). Therefore, we performed subsequent analysis between the pregnant patients (COVID-19 pregnant, n = 20) and female patients with mild cases (COVID-19 non-pregnant, n = 80) to illustrate the effects of pregnancy on SARS-CoV-2 infection. As clinical and immunological features may be different in normal pregnancy, we included a group of healthy pregnant women as control (Healthy pregnant, n = 10) (**Table 2**). Generally, the number and percentage of white blood cells and neutrophils were higher in pregnant women, while the number and percentage of myeloid cells including monocyte, basophil, eosinophil were significantly lower. And pregnant women were more anemic than their non-pregnant counterparts with less red blood cell count, lower hemoglobin level, and lower hematocrit. Laboratory tests also indicated lower levels of creatinine and higher levels of alkaline phosphatase and D-Dimer in pregnancy. The analysis of laboratory findings indicated unique physiological condition of pregnant women, which also explained the difference of clinical features between pregnant and non-pregnant women on SARS-CoV-2 infection. Notably, pregnant patients with COVID-19 had significantly higher levels of C-reactive protein (CRP) compared to the non-pregnant Covid-19 patients and healthy pregnant patients, reflecting an enhanced immune vigilance against infection during pregnancy.

**Table 1:** Comparison of clinical characteristics between pregnant and non-pregnant COVID-19 patients.

Clinical Characteristics	COVID-19 Non-pregnant (n = 299)	COVID-19 Pregnant (n = 20)	P value
**Age, median (IQR), years**	36.00 (31.00–41.00)	33.00 (30.00–34.75)	**0.007**
**Comorbidity, no. (%)**			
Hypertension	16 (5.4)	0 (0)	0.610
Diabetes	11 (3.7)	0 (0)	1.000
Cardiovascular diseases	1 (0.3)	0 (0)	1.000
Chronic bronchitis	0 (0)	1 (5.0)	0.063
Malignancy	10 (3.3)	0 (0)	1.000
**Symptoms on admission, no. (%)**			
Fever	205 (68.6)	15 (75.0)	0.547
Cough	168 (56.2)	8 (40)	0.159
Expectoration	113 (37.8)	6 (30)	0.485
Dyspnea	61 (20.4)	3 (15)	0.775
Diarrhea	47 (15.7)	5 (25)	0.343
Fatigue	51 (17.1)	2 (10.0)	0.547
Chest tightness	40 (13.4)	3 (15.0)	0.741
Chills	29 (9.7)	1 (5.0)	0.706
Myalgia	26 (8.7)	1 (5.0)	1.000
Nausea	30 (10.0)	1 (5.0)	0.706
**Complication, no. (%)**			
Urinary system injury	39 (13.0)	0 (0)	0.149
Liver function injury	3 (1.0)	0 (0)	1.000
**Severity of illness, no. (%)**			**<0.001**
Mild/General	80 (26.8)	20 (100)	
Severe	135 (45.2)	0 (0)	
Critical	84 (28.1)	0 (0)	
**CT Positive, no. (%)**	277 (92.6)	20 (100)	0.379
**Oxygen treatment, no. (%)**	213 (71.2)	17 (85.0)	0.184
**Mechanical ventilation, no. (%)**	12 (4.0)	0 (0)	1.000
**ICU admission, no. (%)**	79 (26.4)	0 (0)	**0.005**
**Onset to admission, median (IQR), days**	11.00 (7.00–20.00)	8.00 (4.25–16.00)	0.073
**Hospitalization, median (IQR), days**	15.00 (10–23.00)	16.50 (11.25–28.00)	0.559
**Mortality, no. (%)**	7 (2.3)	0 (0)	1.000

IQR, interquartile range; CT, Computerized X-ray tomography; ICU, intensive care unit. Bold P values indicated statistical differences.

**Table 2:** Laboratory findings between pregnant and non-pregnant COVID-19 patients and healthy pregnant women.

Laboratory Findings	COVID-19 Non-pregnant (n = 80)	COVID-19 Pregnant (n = 20)	Healthy Pregnant (n = 10)	*P* value
Age, years	34.00 (30.00–39.75)	33.00 (30.00–34.75)	34.00 (29.75–34.25)	0.426
WBC, *10^9/L	6.21 (4.62–7.18) a	7.85 (5.94–9.14) b	8.29 (7.44–10.59) b	**<0.001**
Lymphocytes, *10^9/L	1.77 (1.30–2.23) a	1.33 (0.94–1.60) b	1.35 (1.20–1.80) ab	**0.004**
Lymphocytes, %	0.31 (0.25–0.38) a	0.19 (0.13–0.22) b	0.17 (0.12–0.25) b	**<0.001**
Monocytes, *10^9/L	0.46 (0.35–0.56)	0.48 (0.34–0.63)	0.50 (0.41–0.63)	0.559
Monocytes, %	0.08 (0.06–0.09) a	0.06 (0.05–0.08) b	0.06 (0.04–0.10) ab	**0.016**
Neutrophils, *10^9/L	3.43 (2.38–4.31) a	5.60 (4.32–7.54) b	6.08 (4.93–9.05) b	**<0.001**
Neutrophils, %	0.58 (0.51–0.64) a	0.75 (0.70–0.83) b	0.76 (0.65–0.83) b	**<0.001**
Eosinophil, *10^9/L	0.07 (0.04–0.13) a	0.02 (0.00–0.05) b	0.03 (0.01–0.08) ab	**<0.001**
Eosinophil, %	0.01 (0.006–0.02) a	0.0022 (0.00–0.0071) b	0.0039 (0.001–0.0092) b	**<0.001**
Basophil, *10^9/L	0.02 (0.01–0.03)	0.01 (0.01–0.02)	0.02 (0.01–0.03)	0.185
Basophil, %	0.0031 (0.0021–0.0048) a	0.0017 (0.0011–0.0033) b	0.0023 (0.0011–0.0029) ab	**0.005**
RBC, *10^9/L	4.18 (3.98–4.39) a	3.74 (3.46–4.16) b	3.73 (3.60–3.96) b	**<0.001**
Hematocrit, %	36.90 (35.20–38.70) a	33.25 (31.53–35.70) b	34.10 (30.78–35.60) b	**<0.001**
Hemoglobin, g/L	126.00 (120.00–132.00) a	112.50 (101.50–125.75) b	117.5 (97.75–122.25) b	**<0.001**
Platelet, *10^9/L	231.00 (200.00–288.75)	196.50 (141.50–246.50)	224.50 (160.00–266.75)	**0.042**
ALT, U/L	12.00 (9.00–30.00) a	12.00 (8.25–19.00) ab	8.00 (6.75–12.25) b	**0.036**
AST, U/L	17.00 (14.00–23.00)	17.00 (14.25–22.00)	17.5 (14.5–20.0)	0.866
Total Bilirubin, mmol/L	6.60 (4.40–8.45)	6.15 (5.03–8.08)	5.70 (2.58–8.03)	0.342
Direct Bilirubin, mmol/L	2.80 (2.20–3.60)	3.15 (2.48–3.88)	2.55 (1.60–3.20)	0.144
Indirect Bilirubin, mmol/L	3.80 (2.33–4.98)	2.85 (1.83–4.03)	2.90 (0.98–5.20)	0.065
Serum Creatinine, umol/L	55.00 (50.00–62.00) a	48.00 (42.25–53.75) b	44.50 (36.25–53.00) b	**<0.001**
Blood Urea, umol/L	3.40 (2.70–4.30) a	2.88 (2.18–3.60) b	3.20 (2.54–3.73) ab	**0.046**
Lactic dehydrogenase, U/L	180.00 (149.00–220.00)	192.00 (168.50–247.50)	183.50 (150.25–215.00)	0.365
Alkaline phosphatase, U/L	50.00 (42.00–59.00) a	107.50 (75.50–122.25) b	114.0 (79.25–151.75) b	**<0.001**
Prothrombin time, s	13.50 (13.10–13.50) a	13.10 (12.73–13.48) b	12.9 (12.7–13.15) b	**0.001**
APTT, s	39.70 (37.35–41.58) a	38.50 (33.20–40.05) ab	33.90 (30.55–36.60) b	**0.001**
D-Dimer, ug/ml	0.26 (0.22–0.55) a	1.31 (0.72–1.71) b	1.16 (0.85–1.98) b	**<0.001**
Hs-CRP, mg/L	1.30 (0.45–8.05) a	15.90 (2.58–36.35) b	2.6 (1.2–20.8) ab	**0.001**
Procalcitonin, ng/ml	0.05 (0.04–0.06)	0.06 (0.38–0.10)	0.07 (0.04–0.58)	0.328

The data was shown as median and interquartile range.

WBC, White blood cell; RBC, Red blood cell; ALT, Alanine aminotransferase; AST, Aspartate aminotransferase; APTT, Activated partial thromboplastin time; Hs-CRP, High sensitivity C-reactive protein; TNF, Tumor necrosis factor. *represents multiplication sign. ^a, b, ab^indicated statistical differences between groups labeled with different letters and no significance between groups labeled with the same letter. Bold P values indicated statistical differences.

### Cytokines, Chemokines, and Growth Factors of Pregnant and Non-Pregnant COVID-19 Patients

**Figure 2:**
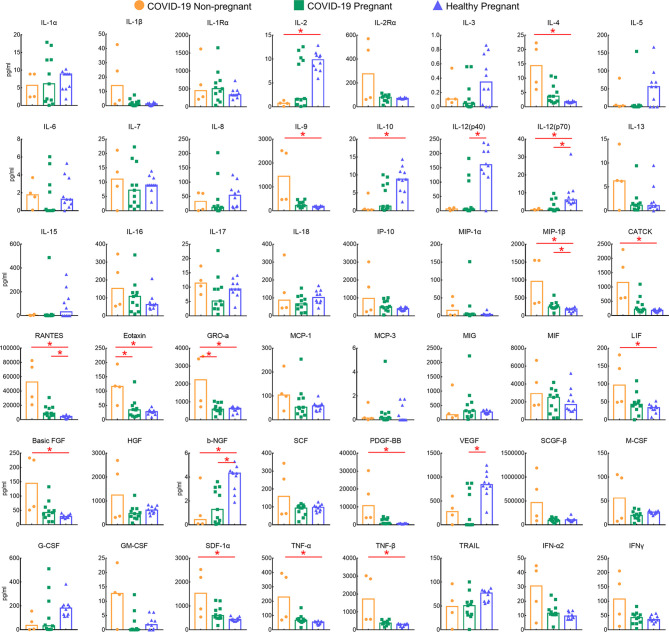
Comparison of plasma levels of 48 cytokines, chemokines, and growths factors between pregnant (n = 11) and non-pregnant groups (n = 4) COVID-19 patients and healthy pregnant patients (n = 10). Red lines and * above the columns indicated significant differences between the two columns.

To investigate the immune response of pregnant women with COVID-19 infections, a total of 11 pregnant patients and four non-pregnant patients with mild cases were compared for cytokines, chemokines, and growth factors (CCGFs). Healthy pregnant women were included as control (n = 10). Clinical characteristics were shown in **Table 3**, and no differences were observed in age, comorbidities, and complications. Healthy pregnant women showed high levels of interleukins including IL-2, IL-5, IL-10, and IL-12, as well as high levels of growth factors such as b-NGF and VEGF, which were largely decreased in COVID-19 pregnant and non-pregnant patients. The non-pregnant COVID-19 patients exhibited high levels of IL-4 and IL-9, and several inflammatory chemokines and growth factors including MIP-1β/CCL4, CATCK/CCL27, RANTES/CCL5, Eotaxin/CCL11, GRO-α/CXCL1, LIF, FGF, PDGF-BB, SDF-1α, and tumor necrosis factors (TNFs) were also highly expressed in non-pregnant COVID-19 group. However, the levels of these CCGFs in pregnant women with COVID-19 were not as high as non-pregnant COVID-19 patients, which may be attributed to the initial low levels of them as exhibited in healthy pregnant women (**Figure 2****)**. Our results indicated that the state of immunomodulation during pregnancy may protect pregnant COVID-19 patients from suffering from cytokine storm and progressing to acute respiratory distress syndrome.

**Table 3:** Comparison of clinical characteristics between pregnant and non-pregnant patients tested for cytokines, chemokines, and growth factors.

Clinical Characteristics	COVID-19 Non-pregnant (n = 4)	COVID-19 Pregnant (n = 11)	Healthy Pregnant (n = 10)	*P* value
**Age, median (IQR), years**	35 (28.25–41.00)	30.00 (29.00–34.00)	34.00 (29.75–34.25)	0.494
**Comorbidity, no. (%)**				
Hypertension	0	0	0	–
Diabetes	0	0	0	–
Cardiovascular diseases	0	0	0	–
Chronic bronchitis	0	1 (9.1)	0	0.515
Malignancy	0	0	0	–
**Complication, no. (%)**				
Urinary system injury	0	0	0	–
Liver function injury	1 (25)	0	0	0.065

IQR, interquartile range.

### Lymphocyte Subsets in the Peripheral Blood of Pregnant and Non-Pregnant COVID-19 Patients

**Figure 3:**
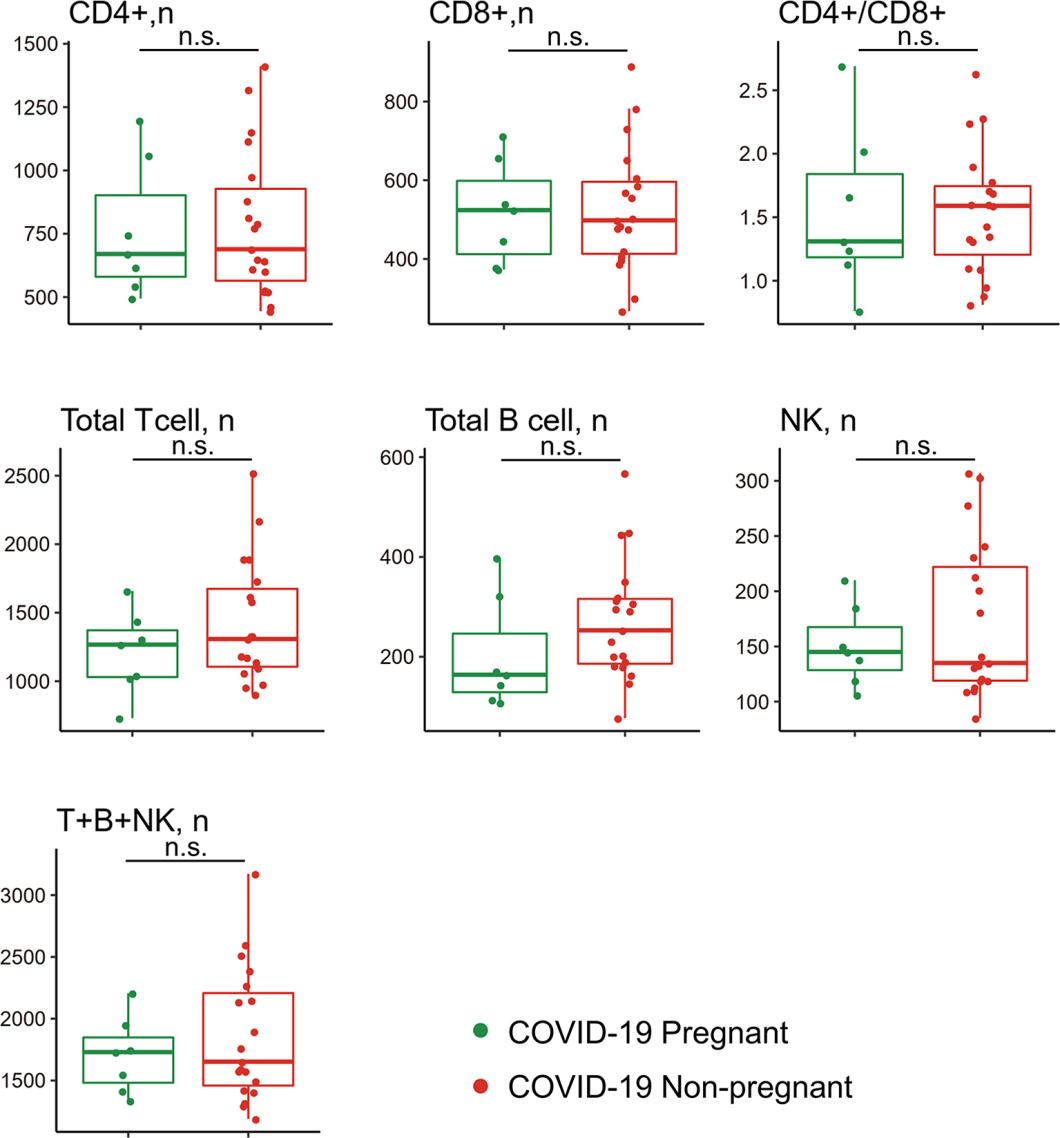
Comparison of peripheral lymphocyte subsets between pregnant and non-pregnant patients with COVID-19. Pregnant COVID-19 group (n = 7); non-pregnant COVID-19 group (n = 19). n.s., no significance.

Given that lymphocyte subsets were reported to be associated with COVID-19 severity and progression, we next analyzed lymphocyte composition in the peripheral blood of the pregnant group (n = 7) and non-pregnant group (n = 19) in COVID-19 patients (**Figure 3**). Albeit no statistical difference was found in clinical characteristics (**Table 4**) and lymphocyte subsets, higher counts of CD3+CD8+ T suppresser (TS) cells were observed in pregnant patients. Correspondingly, pregnant patients exhibited a lower CD4+/CD8+ ratio. In addition, NK cell showed slight high levels among pregnant group. Pregnant patients also exhibited trends of low CD19+ B cell counts, which was consistent with previous reports (22).

**Table 4:** Comparison of clinical characteristics between pregnant and non-pregnant COVID-19 patients tested for lymphocyte subsets.

Clinical Characteristics	COVID-19 Non-pregnant (n = 19)	COVID-19 Pregnant (n = 7)	P value
**Age, median (IQR), years**	32 (29–41)	30 (27–34)	0.139
**Comorbidity, no. (%)**			
Hypertension	1 (5.3)	0	1.000
Diabetes	1 (5.3)	0	1.000
Cardiovascular diseases	0	0	–
Chronic bronchitis	0	0	–
Malignancy	0	0	–
**Complication, no. (%)**			
Urinary system injury	0	0	–
Liver function injury	2 (10.5)	0	1.000

IQR, interquartile range.

## Discussion

To investigate the immunological response of COVID-19 on patients during pregnancy, we performed a clinical analysis as well as CCGFs and lymphocyte subsets analysis of pregnant patients and non-pregnant patients. Our study showed deceased levels of inflammatory cytokines and chemokines of pregnant patients which may contribute to the mild symptoms experienced by these patients.

In our study, pregnant patients with COVID-19 displayed neutrophilia, lymphopenia, and high levels of CRP as well as D-dimer compared with non-pregnant COVID-19 patients, which is in consistent with previous evidence indicating high CRP and D-Dimer, leukocytosis, and elevated neutrophil ratio are more common in the COVID-19 infected pregnant women (23). However, our results also revealed that these physiological findings are typical of normal pregnancy due to adaptations to gestation (24), which may influence the clinical outcomes of pregnant women suffering from SARS-CoV-2 infection.

To investigate the immune status of pregnant patients with COVID-19, we further compared the cytokines, chemokines, and growth factors levels among pregnant COVID-19 group, non-COVID-19 group, and healthy pregnant group. Unlike increased levels of TNFα, IL-6, IL-8, IL-10, CXCL8, and IP-10 observed in H1N1 infected pregnant patients (25), COVID-19 pregnant patients showed relatively lower expressions of pro-inflammatory and anti-inflammatory cytokines. Specifically, macrophage chemokines, which are always referred to as major components of “cytokine storm” during coronaviruses infection (26, 27), including MIP-1α, CTACK, RANTES, Eotaxin, GRO-α, and TNF, showed significant low levels in pregnant patients. Meanwhile, growth factors, such as basic FGF, LIF, granulocyte colony stimulating factor (G-CSF), and PDGF-BB also showed significant low expression in pregnant group. By comparing with healthy pregnant women, we postulated that the immune adaptations during pregnancy may serve as the unique immune response to SARS-CoV-2. It was reported that the immunological response during pregnancy has a shift from a pro-inflammatory state in early stages to an anti-inflammatory state throughout the rest of pregnancy (28). Therefore, in pregnant women who already have dominant anti-inflammatory immunity, the parallel activation of pro-inflammatory and anti-inflammatory immunity in SARS-CoV-2 infection may not lead to pro-inflammatory cytokine storm (19, 29). The high levels of IL-5 and IL-10 (anti-inflammatory) and the low levels of pro-inflammatory cytokines, chemokines, and growth factors observed in our study have validated this conclusion to some extent. Taken together, cytokines response was suppressively regulated in pregnant women following SARS-CoV-2 infection, which may differentiate pregnant COVID-19 patients from patients who suffered from cytokine storm and progressed to acute respiratory distress syndrome (30).

SARS-CoV-2 infection is known to induce a decrease in CD4+ and CD8+ T cell and NK cell levels, more evidently in critically ill patients (31, 32). Therefore, we made comparison of lymphocyte subset counts in pregnant and non-pregnant COVID-19 patients. Compared with non-pregnant patients, pregnant patients showed no significant change of lymphocyte subsets in response to SARS-CoV-2 infection. However, we observed slightly increase number of CD8+ T cells and NK cells in pregnant patients with COVID-19. It is well known that both NK cells (the innate immunity) and CD8+ cytolytic T cells (the adaptive immunity) can destroy the virus-infected cells (33). And numerous clinical reports have shown that the decreased frequency of lymphocytes in the peripheral blood, including CD4+ and CD8+ T cells and NK cells, is closely related to disease severity of COVID-19 (34, 35). In addition, there is a negative relationship between high levels of cytokines and lower T cells in severe COVID-19 patients (4). Enhanced NK and CD8+ T cells response and low levels of CCGFs may protect pregnant patients from progressing to severe cases.

Our study has several limitations. First, the sample size was small because hospitalized pregnant patients accounted only for a small fraction of the sample. Second, cytokine levels may change over time, and because of the limitation of blood samples, our results may only represent a snapshot of these changes. Finally, because this was a retrospective study, the detection time of cytokines was not exactly matched.

In conclusion, our study revealed different immune responses between pregnant and non-pregnant COVID-19 patients. Pregnant patients showed intricately regulated immune response after SARS-CoV-2 infection characterized by a low expression of inflammatory cytokines and a slight increase of NK cell counts. Our study sheds light on the immunological response of pregnant COVID-19 patients, and it has significant implications for immunotherapy for COVID-19. Limited by the retrospective nature of our study, more laboratory evidence is expected to analyze the mechanisms underlying immunoregulation of SARS-CoV-2 to help improve the prognosis of severe cases and reduce mortality.

## Data Availability Statement

The data analyzed in this study is subject to the following licenses/restrictions: The dataset was obtained from Tongji Hospital by approval and are not available to the public. Requests to access these datasets should be directed to tjkeke@126.com.

## Ethics Statement

The studies involving human participants were reviewed and approved by the Ethical Committee of Tongji Hospital, Tongji Medical College, Huazhong University of Science and Technology (TJ-IRB2020401). Written informed consent to participate in this study was provided by the participants’ legal guardian/next of kin.

## Author Contributions

GC collected the data, wrote, and revised the manuscript. QL analyzed the data, wrote and revised the manuscript. JA wrote and revised the manuscript. BY, HB, FL, and JC wrote and drew the figures. YC, HL, and KL designed and supervised the research. All authors contributed to the article and approved the submitted version.

## Funding

This study was funded by the National Key Research and Development Program (2019YFC1005200, 2019YFC1005202), and the Hubei Province Health and Family Planning Scientific Research Project (WJ2019M127).

## Conflict of Interest

The authors declare that the research was conducted in the absence of any commercial or financial relationships that could be construed as a potential conflict of interest.
